# Quantum Biology Using Ultrafast Integrative and Molecular Engineering Tools: A Perspective

**DOI:** 10.34133/csbj.0024

**Published:** 2026-04-07

**Authors:** Krishna Prasad Khakurel, Gustavo Fuertes, Gabriel Žoldák

**Affiliations:** ^1^ Extreme Light Infrastructure ERIC, Za Radnici 835, 25241 Dolni Brezany, Czechia.; ^2^ Laboratory of Biomolecular Recognition, Institute of Biotechnology of the Czech Academy of Sciences, Prumyslova 595, 25250 Vestec, Czechia.; ^3^ Faculty of Science, Pavol Jozef Šafárik University in Košice, Park Angelinum 19, 040 01 Košice, Slovakia.

## Abstract

Quantum biology is the study of how quantum-mechanical effects influence living systems, and hence, it is an advanced interdisciplinary field that integrates biology, physics, chemistry, and mathematics. While the belief that quantum phenomena such as entanglement, tunneling, and coherence exist only in the domain of physical sciences persisted for a long time, recent evidence suggests that their presence in biology should not be overlooked. In the past decade, there have been important advancements in ultrafast structural techniques, such as x-ray free-electron lasers and ultrafast electron diffraction (scattering), which complement optical spectroscopy methods. Historically, ultrafast optical spectroscopy has played a key role in unraveling the mysteries of quantum biology. In this context, integrative ultrafast methods should be utilized to demonstrate and validate quantum events in biology. Furthermore, we emphasize that recent advancements in protein structure prediction and engineering using machine learning can identify and test quantum effects in biology.

Box 1.  Glossary

1. Tunneling: Finite probability of having a particle in a classically forbidden region
2. Coherence: The ability of a quantum system to exist in multiple states simultaneously
3. Entanglement: Nonseparable joint quantum state with correlations not explainable by classical probability distributions
4. Vibronic coherence: Coherences arising from mixing between electronic and vibrational states
5. Decoherence: The process by which a quantum system loses its superposition and coherence due to interactions with the surrounding environment
6. Radical pairs: Short-lived intermediates consisting of 2 simultaneously created radicals (atoms/molecules with unpaired electrons) whose spins are often quantum entangled
7. Open quantum systems: A quantum mechanical system that interacts with an external environment
8. Ultrafast methods: All optical, x-ray, and electron-based methods that can probe processes shorter than sub-100 ps
9. Protein engineering: An approach in biotechnology that aims at designing novel proteins or modifying the existing ones to modulate the performance of the protein
10. Quantum bits, or qubits: The fundamental units of information in quantum computing, unlike classical bits that are strictly 0 or 1.

## Introduction

The development of early 20th-century physics shook the world, leading to 2 distinctly different regimes of the physical world: one governed by the principles of quantum mechanics and the other explained by classical or Newtonian mechanics. The world of living systems could not remain unaffected by these remarkable developments in quantum mechanics. Soon after these new theories emerged, their developers began to consider their implications for biology. The book *What Is Life?* by one of the founding figures of quantum mechanics remains a celebrated read in the field [1]. However, even after a century of development in quantum theory, the application of quantum physics to biological systems is still not widely accepted by a large number of both physicists and biologists. Despite this philosophical resistance, there has been significant progress in identifying instances where quantum principles are essential to explain biological phenomena [2–10]. These phenomena range from tunneling in enzyme catalysis to the use of entangled spin states of radical pairs that interact with a magnetic field by migrating Birds [11,12].

Many quantum events are short-lived [13,14]. The wave nature of a particle collapses as soon as it is localized [15,16]. Coherence in a quantum system decays quickly as it comes to the wet and noisy environment of biological setting [17]. Protons hop from one site of a molecule to another or to a completely different molecule within time scales on the order of picoseconds or shorter [18]. The nature of time-dependent interactions when a molecule entangles with another physical entity remains an open question. This includes the nature of origin of the entanglement process, their evolution in time, and its decay when the coherence is lost. While some of these questions have been explored using ultrafast optical methods and other structural tools independently, it is high time for an integrative ultrafast approach exploiting recent developments in ultrafast x-ray and electron science [19,20] to be used to develop a comprehensive understanding of the quantum phenomena observed in biology. This process of knowledge building can also be greatly facilitated by current advancements in protein structure prediction and engineering using machine learning tools [21,22]. The development of machine learning has also enabled the computation of first-principle details of electron and nuclear dynamics within the entire protein [23]. Such progress can undoubtedly contribute to the development of new experimental strategies and the validation of experimental observations that support the presence of phenomena in biology that can only be explained by the theories of quantum physics.

Quantum biology is conceived and practiced for almost 8 decades. Various experiments have established key concepts in quantum biology. The long-range and short-range tunneling of electrons in photosynthesis has been widely accepted. Kinetic isotope effect (KIE) to assess the probability of tunneling of light atoms in macromolecular systems has served as a gold standard. Coherence and entanglement have been explored in the biological setup. Yet, we are far from understanding the origins of these phenomena. How they exist in the wet and noisy biological environment and contribute to the efficient operation of the molecular machines is less known. Questions such as tunneling of double protons in the DNA during tautomerization still remain unanswered. Integration of the matured ultrafast spectroscopic tools and evolving structural tools including the artificial intelligence (AI)-based macromolecular engineering can prove to be significant in answering some of these questions.

## Evidence of Quantum Phenomena in Biology

Life science is a rich discipline within the natural sciences, where often delicate phenomena are studied. While numerous textbook theories in chemistry and physics can be tested using biological objects, there is only a handful of example supporting phenomena where quantum mechanics may provide a possible explanation. Some of these observations in quantum biology can be grouped into the following categories:

### Tunneling of electrons and light atoms

Electron transfer reactions have a crucial importance in many biological processes, such as activation of sensory proteins [24], DNA ultraviolet-damage repair [25], energy harvesting [26], and sensing of magnetic fields [27]. A foundational study by De Vault and Chance in 1966 [28] marked the beginning of experimental explorations into these reactions. Specifically, they investigated the kinetics of oxidation of cytochrome proteins in photosynthetic bacteria. Two kinetic components were found: a temperature-dependent fast component following Arrhenius kinetics and a nearly temperature-independent slow component. These experiments laid the basis for the concept of electron and nuclear tunneling in biology [29].

This concept of tunneling has since become a widely accepted explanation for the transport of subatomic particles, notably electrons and protons, which are integral for life’s energy processes. In 1963, Löwdin [30] proposed that protons in hydrogen bonds of DNA could tunnel, thereby altering bond lengths and transforming base pairs from their canonical to tautomeric forms. Quantum tunneling in biology has set the stage for understanding various biological phenomena, including electron transport in photosynthesis [31,32], cellular respiration [33], as well as in DNA and enzyme-catalyzed reactions [34].

**Fig. 1.**
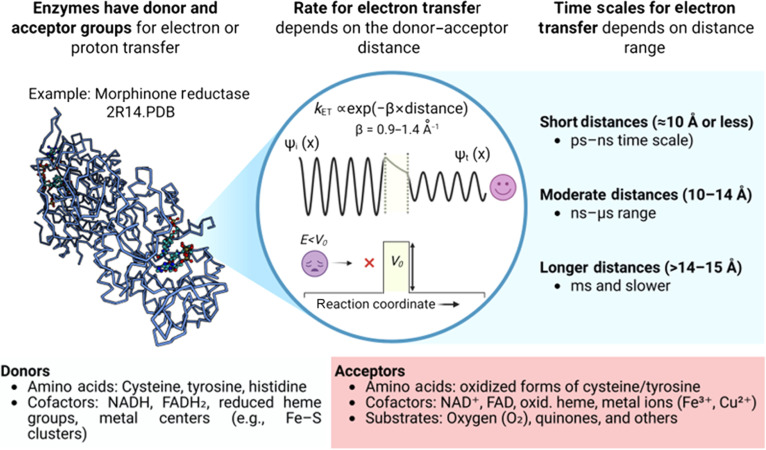
A schematic of tunneling in enzymes and the corresponding time scales. NADH, reduced nicotinamide adenine dinucleotide; FADH2, flavin adenine dinucleotide (reduced form); NAD, nicotinamide adenine dinucleotide; FAD, flavin adenine dinucleotide.

The phenomenon of proton tunneling, although speculated earlier, has found experimental support in 1989 with studies on alcohol dehydrogenase, an enzyme that transfers protons in metabolic reactions [35]. Subsequently, it was shown that quantum tunneling in enzymes is influenced by protein dynamics, which modulate distances between proton donors and acceptors and affect the electrostatic environment of active sites [36]. Quantum tunneling has implications for enzyme catalysis, inhibition, and design (Fig. 1) [37,38]. Quantum tunneling is sensitive to the mass of a particle, and replacing hydrogen with deuterium, which has double the mass, affects tunneling rates, a phenomenon known as the KIE. This effect has been confirmed in various enzymatic reactions attributed to proton tunneling [39]. Multiple proton exchanges in cyclic molecular structures and heavy-atom tunneling in biomolecular reactions [40] exemplify the extent of quantum tunneling. Overall, our understanding of quantum tunneling in biological systems has evolved significantly since the initial experiments, providing a deeper understanding of the molecular mechanics of fundamental life processes.

The time scale of electron tunneling in proteins ranges from femtoseconds to nanoseconds depending on the tunneling distance and the environment. Similarly, the tunneling time scales for protons are also in the range of picoseconds and subpicoseconds. The time scale for heavy-atom tunneling in proteins is rarely reported, but drawing the knowledge from the smaller organic molecules, this time scale also ranges in picoseconds. Hopping of the particle from one site to another is not merely a translocation of the particle; several structural dynamics of the nearby sites are involved in this process, understanding of which demands an integrated ultrafast method. While the concept of electron and light-atom tunneling in macromolecular systems is established and has been supported by some experimental evidence, viewing the molecular movie of such process and proving their existence in biological environments without any bias is still to be realized.

### Entanglement

Entanglement is one of the fundamental quantum mechanical phenomena that describes nonseparable joint quantum states with correlations not explainable by classical mixtures. Quantum entanglement has been observed in biological settings on several occasions. The mechanistic details of the origins of the entanglement and how it collapses are largely unexplored.

Quantum entanglement between the electron clouds of nucleic acids in DNA was studied by Rieper et al. [41] using a model in which electron clouds of the nucleic acids were viewed as a chain of coupled quantum harmonic oscillators. Experiments where living sulfur bacteria are entangled with a quantized field of light have been reported in [42], which also serves as proof that quantum event can be present in wet and noisy environments of biological settings. Proposed models of entanglement involving tubulin in microtubules and their possible relevance to consciousness have also been discussed [43,44]. Dynamic entanglement in the biological environment [45] and heat capacity as an indicator of entanglement [46] are a few examples of the entanglement expected in biology. The probability of entanglement in photosystem complexes has been discussed rigorously in [14] where probability of multipartite entanglement is discussed to be considerable up to few picoseconds.

**Fig. 2.**
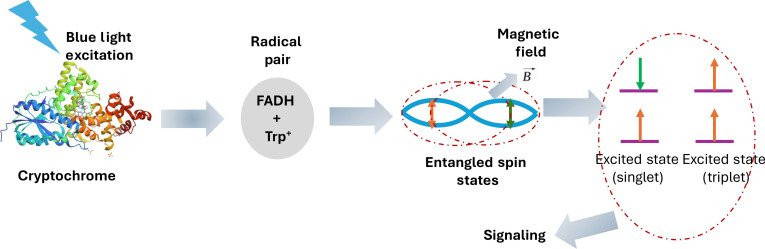
A schematic of entangled spin state in cryptochrome protein upon photoexcitation during magnetoreception. FADH, flavin adenine dinucleotide (reduced form).

In order to render a complete picture of how the entanglement starts, how the exchange/sharing of the information takes place, and how such information is retained, one needs to probe all the mechanistic details of the process in addition to the dynamics of electronic states. Especially when the entangled particles are complex and propagation/storing of the information can potentially be through multifold structural and electronic restructuring, the use of integrative ultrafast tools can bring up new insights of the phenomenon. An instance of such coupled structural changes and potential involvement of the spin entanglement is predicted to occur during magnetoreception in cryptochrome photoreceptors. We show a schematic of the flow of the dynamic event during signaling in Fig. 2, where the evolution of the entangled state occurs through not only electronic/vibrational dynamics of the chromophore (flavin adenine dinucleotide) but also through the dynamics in the chromophore and the residues in the vicinity.

### Decoherence

**Fig. 3.**
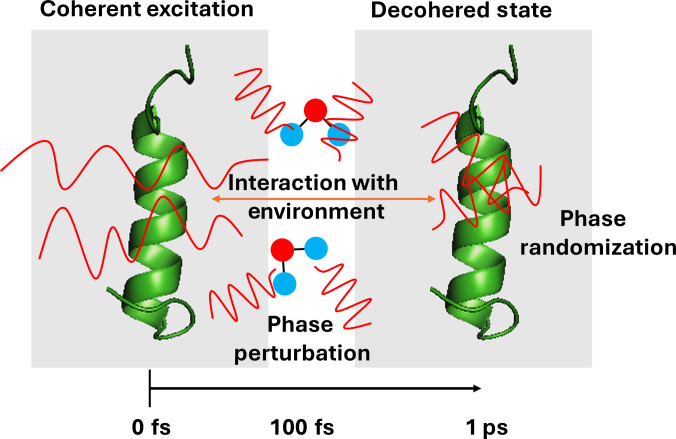
A schematic of loss of vibrational/electronic coherence in proteins and the associated time scales.

Decoherence is another quantum mechanical phenomenon that typically takes place in the femtosecond to picosecond time scales (Fig. 3), depending on the surrounding environment. The loss of phase coherence when perturbed by external factors is typically termed decoherence in quantum mechanics. In general terms, it is the loss of information retained by any system. In physical systems where the experimental factors can be controlled, decoherence has been measured and decoherence time has been quantified. In wet and noisy conditions present in the biological environment, decoherence is supposed to be short-lived and is sparsely characterized. There are only a few instances where decoherence in a biological entity has been measured and predicted to influence the biological function of the molecules [47–50]. Experimental evidence of long-lived decoherence in several photosynthetic complexes has triggered a question on the origin of such decoherences [51]. Coherence in the neuronal system and the relevant time scales and contributing factors have been previously discussed [52]. Here, by decoherence, we refer to the loss of electronic/vibronic coherences and excitonic coherences when the biological object entangles with the environment. Further, it is to be noted that the time scale of decoherence is system dependent and longer decoherence and different types of decoherence time can be expected for different systems. The time scale shown in Fig. 3 is relevant to vibrational or electronic decoherence.

While decoherence typically takes place in the femtosecond to picosecond time scales (Fig. 3), instances where decoherence lasts for longer time up to microseconds have also been reported in a few occasions [53,54]. In proteins, the decoherence channel is often accompanied by the change in atomic positions, vibrational motions, local side chain movements, reorganization of the solvent molecules, etc. [55]. To render a complete picture of the decoherence process in proteins, ultrafast structural tools integrated with the optical spectroscopies are a must.

The quantum phenomena discussed above have been reported in one or another form in biology. While some of them have been identified to be indispensable for biological activity, most are still argued not to be functionally relevant. Dissecting the mechanistic details of these phenomena and correlating their relationship with the biological function is needed to prove or refute whether the best theory explaining the physical world is also of relevance to the biological world.

In the following, we will describe some of the exemplary model systems where the quantum phenomena and the interplay of ultrafast dynamics have been discussed. It is beyond the scope of this perspective to get into all the systems where such a phenomenon has either been observed or predicted to occur.

1. Dihydrofolate reductase (DHFR) is a well-studied system that exemplifies quantum tunneling in enzymatic reactions [56]. It has long been discussed that the enzyme utilizes quantum tunneling to facilitate the transfer of a hydride ion during the reduction of dihydrofolate. DHFR structure and dynamics are tightly coupled to the chemical step of hydride transfer. Quantum tunneling is suggested by the observed KIEs, which are significantly higher than those predicted by classical models [57]. Understanding tunneling in DHFR is crucial due to its role in nucleotide biosynthesis and as a target for antibiotics and chemotherapeutic agents. Further, the ultrafast dynamics associated with the hydride transfer has also been hypothesized to be vital for the origin of the enormous gain in the catalytic efficiency [58]. It has also been proposed along this hypothesis that the faster dynamics in DHFR that follows with the hydride transfer builds up for the enormous gain in the catalytic power. In order to decipher the precise mechanistic detail of such processes, using an integrated ultrafast approach is a must to gain an unbiased insight into the phenomenon. Use of integrated methods discussed in this article can potentially visualize the molecular movies of enzyme catalysis including the hydride transfer with atomic resolutions. Such experimental demonstration definitely will provide more comprehensive information of whether or not tunneling/dynamics of hydrides has a role to play in enzyme catalysis. Similar proton tunneling and fast dynamics have also been proposed in other enzymatic systems and DNA.
2. Cryptochrome: Since the 1970s, magnetoreception has been a prime attraction in the field of quantum biology [59]. Cryptochromes, blue light receptor flavoproteins, are hypothesized to facilitate the magnetoreception in migratory birds and animals [60]. On exposure to the blue light, the flavin in the cryptochrome is photoreduced, leading to the formation of spin-correlated radicals, the so-called radical pairs [61]. Such radical pairs are known to show sensitivity toward weak magnetic fields [62–64]. Following this logic, cryptochromes are supposed to act as a magnetoreceptor molecule through spin-correlated radicals [65,66]. A comprehensive understanding of magnetoreception, including the molecular events from chromophore photoexcitation to spin-correlated radical pair generation, needs an integrated approach to answer the unresolved questions.
3. Respiratory and photosynthetic complexes: Electron transfer is a fundamental process in photosynthesis and respiration [67]. Electron transport is already a well-understood quantum process. However, what remains to be understood is how these processes are related to decoherence and the associated protein dynamics [55]. Typically, electron transfer reactions are fast. Any tool to probe the decoherence and the protein dynamics needs to have a resolution better than 100 fs. The dynamics at this time scale can be probed with a spatial resolution on the atomic scale [68]. Therefore, to understand the interplay between electron transfer, decoherence, and dynamics in proteins, one needs to make use of an integrated approach. Further, entanglement in photosynthetic complexes is supposed to take place in the time scale of subpicoseconds to few picoseconds depending on the environment they are in. The use of the integrated approach discussed in this article can facilitate all coupled structural evolution during this process including the electronic transitions.

## Necessity of the Integrated Approach

Before diving into the advantages of the integrative approach, let us summarize what each technique has to offer to build a comprehensive understanding of quantum biology.

### Ultrafast optical spectroscopy

Optical spectroscopy has been extensively used to probe macromolecular dynamics and provide a bridge between observable world and quantum physics. Over the years, several novel modules of ultrafast optical spectroscopy have evolved [69]. A comprehensive review of all available ultrafast optical spectroscopic tools is beyond the scope of this perspective. Ultrafast spectroscopy has been used in a multitude of applications in biology, ranging from electron transport to probing decoherence in photoexcited systems [70]. Spectroscopic methods have also been used to entangle photons and molecules as a promising approach to generating an entangled photon-pair source despite the protein molecules being in a strongly decohering environment of a room-temperature solution [71]. Transient visible absorption spectroscopy provides large information on the electronic dynamics, while time-resolved vibrational spectroscopy (infrared and Raman) provides information on structural evolution at the bond level. However, these techniques are not able to provide coupled nuclear dynamics and the global structural evolution of the macromolecules.

### Ultrafast x-ray science

X-ray crystallography is among the widely used methods to probe the structural details of macromolecules [72]. With the emergence of ultrashort and ultra intense x-ray pulses, macromolecular crystallography has reached new heights, providing atomic-level views of molecules in action [73]. Visualizing fast processes in chemistry, enzyme catalysis, and macromolecule-aided photo-to-chemical energy conversion has been possible with the new transient x-ray tools, which include solution scattering, crystal diffraction, and spectroscopy. They provide local and global dynamics in the macromolecules on the time scales relevant to the processes where quantum mechanics can play a role.

The past few decades have witnessed not only femtosecond x-rays but also a significant interest in the development of attosecond x-rays, both at longer and shorter wavelengths [74]. While most of the attosecond x-rays have been based on the tabletop extreme ultraviolet high harmonic generation, the recent development of attosecond pulses of hard x-rays at LINAC coherent light source opens new frontiers [75]. With the development of attosecond x-ray sources, it becomes more feasible to track ultrafast electron motions in macromolecules, which could provide more accurate information about several quantum processes in biology.

### Ultrafast electron diffraction

Hydrogen bonds are a significant part of the biomolecular networks [76]. They are critical for how several biological phenomena occur. Many enzyme-catalyzed reactions involve the breakage and formation of hydrogen bonds [77]. Further charge transfer in macromolecular processes is quite common. Often, such charge transfer reactions introduce subtle charge differences in the acceptor/donor sites of the macromolecules. Electron scattering, unlike x-rays, is more sensitive to subtle changes in charges and the position of the light atoms (hydrogen and proton) [78]. In recent cryo-electron microscopy studies of macromolecules, localization of hydrogen/proton, which was often considered challenging in traditional crystallography, is becoming more routine [79]. Probing the dynamics of these light atoms and subtle changes in charges is more feasible with ultrafast electron diffraction/scattering than with x-rays. Evidence of such possibilities has been demonstrated in small organic molecules [80]. Extension of these tools to the macromolecules represents a challenge to overcome in the future.

### AI-informed protein engineering:

Computational tools have been instrumental in predicting and understanding the origins of the quantum phenomenon in biology. With the recent progress in protein structure prediction and quantum calculations in proteins, AI has pushed the horizons of the computational approaches that can be used in the study of protein structure and dynamics. AlphaFold’s success stems from applying transformer-based architectures inspired by NLP (natural language processing) to a biological problem, such as predicting protein 3-dimensional structures [21,81]. However, when analyzing intrinsic quantum phenomena, such as entanglement or tunneling, classical deep-learning models (no matter how advanced) may be complemented by specialized quantum computing and simulation methods that directly model quantum states and interactions. While classical computing can be used in prediction/computation of quantum phenomenon through machine learning approaches [82–84], this can be made more efficient by introducing the capabilities of quantum computing. Large language models and NLP operate on classical data (text tokens) using purely classical computations. In contrast, quantum machine learning must encode, process, or simulate actual quantum states—involving amplitudes, superposition, and entanglement. Hence, large language models do not natively handle the intrinsically quantum features that machine learning algorithms require for accurate modeling of quantum phenomena. Recent development can unfold a new understanding of the interplay of quantum mechanics in biology.

**Fig. 4.**
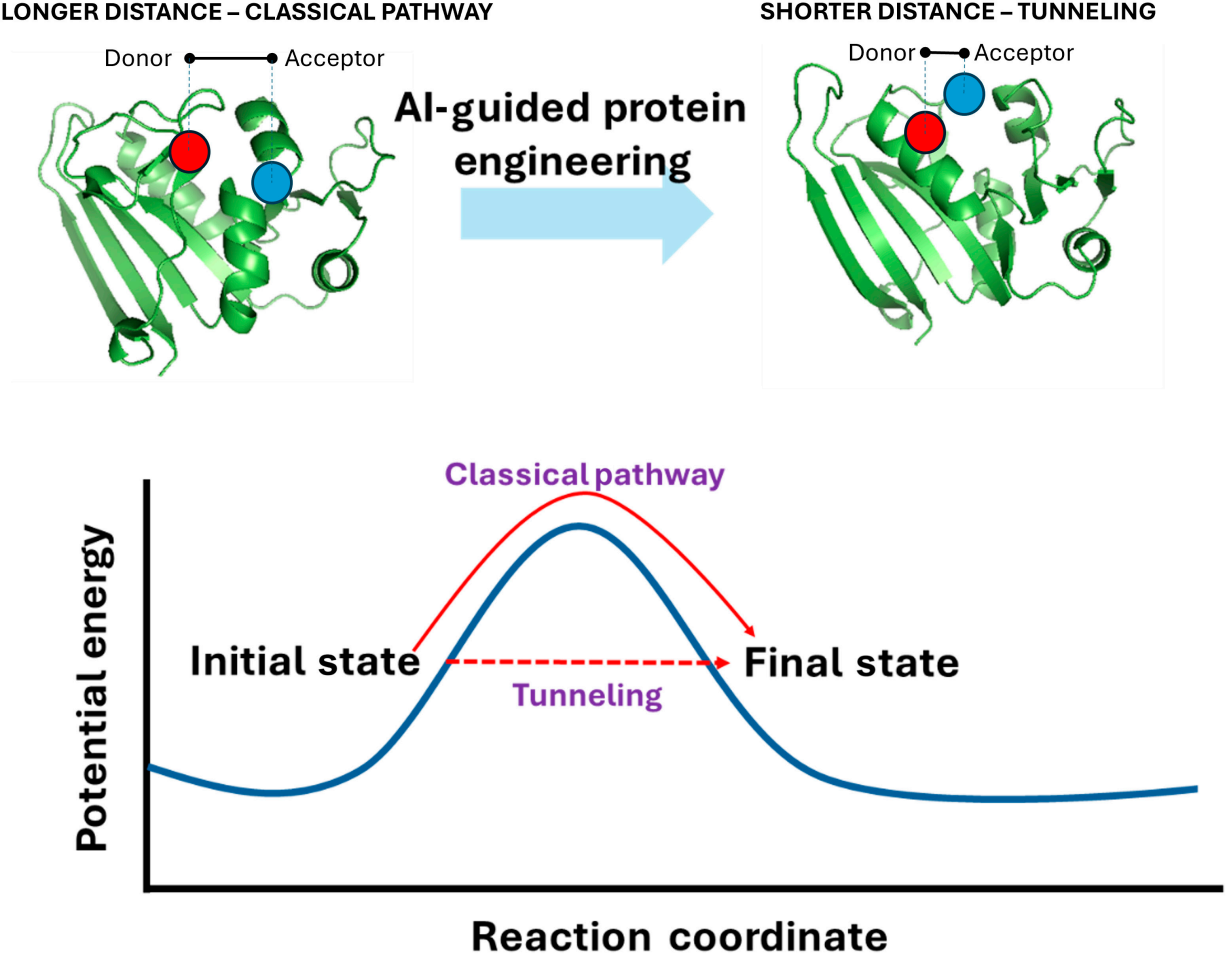
A schematic of protein engineering helping in deciphering/tuning quantum phenomena in biology.

Quantum biology and protein engineering can inform and accelerate each other [85]. AI-based computational protein design can already generate high-affinity binders for a number of biological cofactors and chromophores, like heme and bilins [86]. Moreover, protein engineering is not limited to the 20 canonical amino acids: today, more than 500 noncanonical amino acids, including those optically active, can be routinely incorporated at virtually any desired location to understand the behavior of natural proteins and to create new-to-nature proteins [87,88]. We envision 3 major areas where “quantum proteins” derived from merging quantum biology and protein engineering could provide a wealth of information. First, the modern molecular engineering toolbox could be leveraged to design protein active sites for tunneling. For instance, by positioning proton donor–acceptor residue pairs at varying distances and angles, one could shape the potential energy surface, thereby increasing or decreasing tunneling probabilities (Fig. 4). The system can be made light responsive (and thus amenable to the ultrafast time-resolved techniques mentioned before) through the use of photoreactive noncanonical amino acids as pioneered by Liu et al. [89]. Alternatively, photoacids and photobases, which donate or accept a proton exclusively after light excitation [90], might also be employed as phototriggers. A second exciting research direction would be coherence management in energy/charge transport e.g., by scaffolding chromophores at precise distances and orientations to generate desired excitonic couplings and delocalization lengths. A third area of synergism between quantum biology and protein engineering would be in spin and radical-pair control. This may be achieved (a) by embedding radical-generating cofactors, e.g., flavins [91,92], (b) by placing nuclei with specific spins near radical sites, and (c) by photoinduced electron transfer between appropriately spaced donor–acceptor pairs (similar to the aforementioned cases of excited-state proton transfer). With all these tools in hand, we foresee the creation of magnetoreception-inspired sensors and photocatalytic systems enabling artificial photosynthesis.

While all of these tools, in one form or another, have been applied to probe biological phenomena that potentially involve quantum mechanics, they have rarely been used in an integrated approach to provide a unified picture of these processes. The use of optical spectroscopy to investigate electron tunneling in photosynthesis and x-ray crystallography to gain insights into proton tunneling in enzyme catalysis are among the cases where the independent use of these tools has made significant contributions to our understanding of these phenomena. However, despite decades of study, a conclusive narrative is still lacking. This has often been a major setback in applying quantum mechanics to the interpretation of biological functions. With all the tools available, it is time that an integrative approach exploiting the capabilities of each of them be adopted to build a comprehensive understanding of quantum biology (Fig. 4). In addition to these tools, support from efficient computations is essential to understand the role of quantum mechanics in biology, such as AI-generated density functional theory-equivalent charges and charge dynamics.

**Fig. 5.**
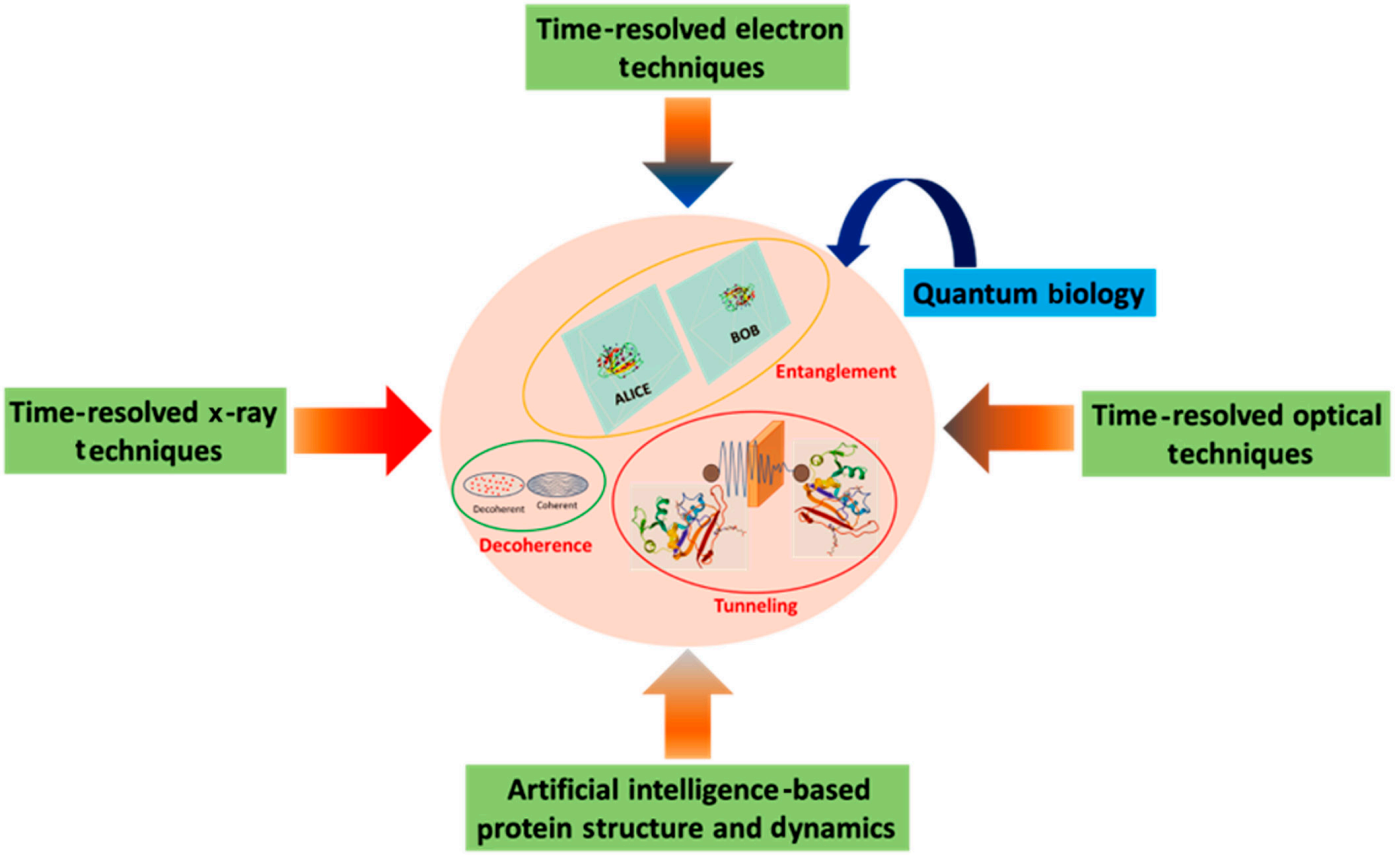
A schematic of the use of integrated methods to probe quantum biology.

An integrated approach to studying these phenomena provides all the possible information necessary to interpret these systems. For instance, when considering proton/hydrogen tunneling in enzymes, all electronic and vibrational dynamics that facilitate the tunneling event can be probed by optical spectroscopic tools. Meanwhile, time-resolved structural probes, such as x-ray scattering and x-ray free-electron laser crystallography, are needed. The localization of protons/hydrogens in most enzyme structures using x-rays is nontrivial, which is where electron probes should be utilized, offering better sensitivity to these light atoms. While time-resolved electron crystallography or microscopy has not yet been developed for macromolecules, these advancements might be realized in the near future. With cryo-electron microscopy tools maturing and evolving in new ways, and significant developments being made in time-resolved electron probes, the next few years will be an exciting period to witness such progress. We believe that these progresses can decipher new understanding in quantum biology and also potentially on the development of a new generation of bio-inspired quantum technologies. A schematic of proposed integrated approach is shown in Fig. 5.

## Mini-Cases for Use of Integrated Methods

1. Photosynthesis (entanglement): Entanglement is closely related to quantum coherence but represents a stronger form of non-classical correlation between quantum states. Reports have been made that entanglement in a photosystem lasts for several hundreds of femtoseconds at room temperature. At low temperatures, there is finite entanglement in the system up to 5 ps [14]. While optical spectroscopy can provide signatures of entanglement indirectly, the structural evolution that accompanies with the evolution of the probability of entanglement is still not understood. An integrative approach of using both ultrafast optical and structural tools can track the evolution of both the nucleus and electrons, which otherwise is not possible.
2. Enzyme catalysis (tunneling): “Promoting protein motions”, i.e., specific fluctuations that might lower the barrier height or promote tunneling by reducing donor–acceptor distances can drive enzymatic reactions. Such models include promoting vibrations, environmentally coupled tunneling, and vibrationally enhanced ground-state tunneling. Several of these proposals suggest that the anomalous temperature and pressure dependences of experimentally observed reaction rates and KIEs are the consequence of protein motions on the pico- to femtosecond time scale that reduce the width and/or height of the potential energy barrier along the chemical reaction coordinate. However, a connection between promoting motions and potential energy barrier modulation has never been demonstrated directly, which is where the integrative methods can come into play.
3. Engineered fluorescent proteins as quantum bits (superposition and entanglement): Recent work demonstrates how a genetically encodable fluorescent protein can be transformed into a fully functional spin qubit, thereby bridging quantum sensing with molecular bioengineering [93]. By leveraging the intrinsic metastable triplet state of enhanced yellow fluorescent protein, it is possible to initialize, control, and read out quantum states across cryogenic and room-temperature regimes. This represents the same conceptual merger central to quantum biology: using ultrafast photophysical pathways as experimentally addressable levers for quantum manipulation. Moreover, because the qubit is encoded directly into living cells, the work highlights how protein engineering can embed quantum-active centers into biological environments, enabling nanoscale sensing, coherent control, and quantum-enabled imaging.
4. Engineered fluorescent proteins as quantum sensors (magnetic field sensitivity): Quantum-mechanical spin phenomena can be engineered directly in cellulo through magnetosensitive fluorescent proteins, so-called MagLOV [94]. These are proteins derived from the large class of light–oxygen–voltage (LOV) photosensory receptors [95], which are typically poorly fluorescent in their native states. This approach combines protein engineering via directed evolution with spin-based optical readouts to create genetically encoded reporters whose fluorescence responds to magnetic and radio frequency fields, enabling quantum-level measurements under physiological conditions. This aligns with the broader perspective of integrating photophysics, radical-pair spin chemistry, and biocompatible quantum sensing, ultimately showing how quantum effects—traditionally probed by ultrafast spectroscopy—can now be intentionally designed, tuned, and utilized within biological systems for multiplexed imaging, environmental sensing, and spatial localization.

## Conclusion

To conclude, we propose that an integrated approach, combining all ultrafast optical and structural probes in parallel with AI-accelerated protein engineering, should be used to probe quantum phenomena in biology. While processes such as electron transfer and decoherence in photosynthesis have been understood, leveraging the capabilities of state-of-the art spectroscopic tools, the associated structural dynamics can only be understood using ultrafast structural tools. Our proposed integrated approach will provide all electronic and nuclear dynamics associated with the quantum phenomena and will introduce the possibility of tuning it through the approach of protein engineering. When it comes to the tunneling of light atoms in enzyme catalysis, the KIE has been a gold standard. However, the KIE provides only an indirect assessment of the tunneling probabilities. In order to ascertain whether light atoms propagate classically or quantum-mechanically, the best way would be to track them in real time structurally. Currently, these capabilities are provided by the ultrafast x-ray and electron diffraction-based methods. We predict that integrating ultrafast tools and protein engineering can provide a more comprehensive picture of tunneling probability as compared to KIE, which can potentially suffer from processes such as vibrational zero-point effects, donor–acceptor distance fluctuations, and proton-coupled electron transfer that can introduce ambiguities. Moreover, protein engineering is needed to gain insight into the processes involved. Recent developments in AI and the molecular engineering toolbox can provide those capabilities and accelerate the design of next-generation “quantum proteins”. This perspective proposes that the joint use of ultrafast structural biology/biophysical methods and protein technologies should be the roadmap for identifying, sensing, and manipulating quantum events in biology.
